# Single electron-photon pair creation from a single polarization-entangled photon pair

**DOI:** 10.1038/s41598-017-16899-w

**Published:** 2017-12-05

**Authors:** Kazuyuki Kuroyama, Marcus Larsson, Sadashige Matsuo, Takafumi Fujita, Sascha R. Valentin, Arne Ludwig, Andreas D. Wieck, Akira Oiwa, Seigo Tarucha

**Affiliations:** 1 Department of Applied Physics, The University of Tokyo, Bunkyo-ku, Tokyo, Japan; 2 Lehrstuhl für Angewandte Festkörperphysik, Ruhr-Universität, Bochum, Germany; 3 The Institute of Scientific and Industrial Research, Osaka University, Ibaraki, Osaka, Japan; 4 Center for Emergent Materials Science, RIKEN, Wako, Saitama, Japan

## Abstract

Quantum entanglement between different forms of qubits is an indication of the universality of quantum mechanics. Entanglement transfer between light and matter, especially photon and spin, has long been studied as the central concept, but it remains technically challenging for single photons and spins. In this paper, we show paired generation of a single electron in a GaAs quantum dot and a single photon from a single polarization-entangled photon pair. We measure temporal coincidence between the single photo-electron detection and the single photon detection. Considering a single photon polarization is converted to an electron spin via an optical selection rule, the present result indicates the capability of photon to spin entanglement transfer. This may be useful to explore the physics of entanglement transfer and also for applications to quantum teleportation based quantum communication.

## Introduction

Entanglement transfer between light and matter has long been studied in order to explore fundamentals of quantum physics^1^. Moreover, this phenomenon is expected to be an important ingredient to realize distributed photonic quantum communication based on quantum repeaters but not yet well exemplified^2,3^. Coherent transfer of single-particle states between different kinds was previously demonstrated for a single photon to a single ion trapped in a Paul trap and Doppler cooled^4^. In addition, entanglement generation between a single photon and a single atom was performed using photon emission from the atom^5,6^. An electron spin or a hole spin confined in a semiconductor quantum dot has been regarded as one of the best candidates to engineer entanglement between a solid-state quantum and a photonic quantum. Indeed, experiments on entanglement generation has been reported for a photon emitted from an InGaAs self-assembled quantum dot (QD) and a remaining electron spin in it^7,8^ and also for an InAs QD and a remaining electron spin^9^. However, direct experimental demonstration of entanglement transfer from photons to solid-state spins or vice versa remains challenging. Achieving this provides more variations of entanglement engineering between distant spins as well as between a photon and a spin spatially separated. Especially projection of single entangled photon pairs to single distant electron spin pairs in nano-scale quantum devices may provide a new tool to study non-local entanglement in mesoscopic solid-state physics.

Quantum state transfer of light to spin in solid^10^ was experimentally demonstrated for ensembles of photon polarization states and electron spin states with a GaAs quantum well^11,12^. For the single-particle states we previously demonstrated angular momentum transfer from polarization of a single photon to spin of a single electron using GaAs QDs^13^. This implies that single polarization-entangled photon pairs can be used as a source to establish photon-spin entanglement and spin-spin entanglement for single photo-electrons trapped by QDs. However, no experiments on such photo-electron generation in QDs have been reported to date. Note the GaAs QDs^14^ may have advantages compared to the self-assembled QDs in the ability of quantum gating^15,16^ and scale-up^17–20^, both of which are needed to combine the quantum communication with gate-based computation, but still challenging in self-assembled QDs.

In this paper, we report on the experimental observation of coincident generation of a photo-electron in a GaAs dot and a photon in a photo-detector from a polarization-entangled photon pair. One of the paired photons is irradiated on the GaAs QD, while the other on a single photon counter. Real-time measurements of the photo-electron in the dot by a charge sensor^21–26^ and the photon by the photon counter are performed simultaneously to identify the temporal coincidence between the photo-electron detection and the photon detection. Although a fast real-time detection technique of an electron has been established for gate defined QDs in recent years, photo-electron detection is still technically difficult because QDs made in a doped quantum well becomes eventually unstable due to charge noise and persistent photo-conductivity when shining light pulses. To minimize such instability we irradiate photons with sufficiently weak intensity. The averaged interval of single photons irradiated on the dot and the photon counter is adjusted to be larger than the time resolution of the charge sensor and much larger than that of the photon counter. Therefore zero to two photons (N = 0 to 2) are mostly detected by the photon counter in a time window for detecting the photo-electron by the charge sensor. However, this does not always guarantee the detected photo-electrons and photons are originated from the same entangled photon pairs. By comparing the probability of detecting zero to two photons between with and without the photo-electron detection we are able to confirm coincident detection of a photo-electron and a photon from the same polarization-entangled photon pair. Because we already know in our previous experiment that a photon polarization is transferred to a spin angular momentum of a photo-electron^13^, the present result provides a critical technical step for creation of an electron spin entanglement induced by a polarization-entangled photon pair and for applications to distributed quantum communication.

## Setup

### Optical setup for Spontaneous Parametric Down Conversion

**Figure 1:**
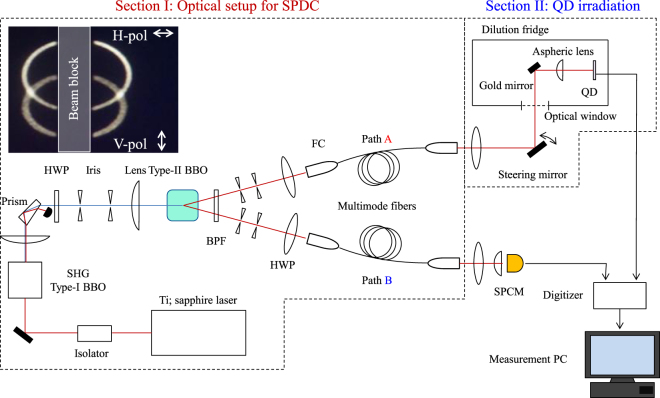
Experimental setup for a single photo-electron-photon pair creation and detection. Schematic of the experiment setup used for the coincidence measurement of the photo-electron excited in the dot and the photon at SPCM. The setup can be separated into two sections, I and II. Section I is an optical setup to generate paired photons by SPDC. A Ti:sapphire ultra-short pulsed laser is used to generate SHG photons with a Type-I BBO crystal. The SHG photons are irradiated on a Type-II BBO crystal to generate SPDC photons after removing the original laser light with a prism and two irises, and rotating the polarization with a half wave plate (HWP) to satisfy the phase matching condition of SPDC. The paired photons are emitted in two different SPDC photon cones through a band pass filter (BPF). A series of irises are placed to cut out the crossing points of the photon cones. After filtering the energy and the spatial position, the photons are coupled to the multimode fibers. The fiber output photons are observed by SPCM. Section II is a setup for irradiating the photons on the dot and detecting the photo-electron. The dilution refrigerator has an optical window at the bottom and the irradiating photons along Path A are focused by an aspheric lens on the sample through this optical window. The position of the irradiating photon spot can be aligned by tilting a steering mirror. Finally, the charge sensing signal of detecting the photo-electrons and the SPCM signal of counting the photons are monitored by the digitizer. The inset is a far-field image of the SPDC photons observed by a near infrared sensitive CCD camera. The upper, and the lower ring indicate the emission of horizontally polarizing photons, and vertically polarizing photons, respectively.

Figure 1 schematically shows the experimental setup consisting of two sections: Section I for generating single photon pairs using a spontaneous parametric down conversion (SPDC) technique^27,28^ and Section II for detecting photo-electrons in the dot and photons by the photon counter upon separate irradiation of single photons from the photon pairs.

A Ti:sapphire ultra-short pulsed laser (MIRA-900P) with auto-correlation width of 3 psec and repetition rate of 76 MHz is used to generate the SPDC photons. The laser pulse has a peak power of 2 kW with the center wavelength of 808 nm (1.5345 eV). This wavelength is adjusted to coincide with the excitation energy for the heavy hole (HH) exciton in the GaAs QD. The photon energy is doubled via second harmonic generation (SHG) using a Type-I Beta-Barium-Borate (BBO) crystal. To extract only the SHG photons a Pellin-Broca prism, several irises and short pass filters are used. The average power of the filtered second harmonic light obtained is about 100 mW. The SHG photons are irradiated on a Type-II BBO crystal in order to generate SPDC photon pairs. A band pass filter whose transmittance is maximal at 800 nm is placed just after the crystal to only transmit the SPDC photon pairs. The paired photons have two kinds of linear polarization orthogonal to each other and are emitted in two different directions. The inset of Fig. 1 shows the far-field image of the emitted photons observed by a CCD camera. The upper and lower ring indicate the horizontally, and vertically polarized photons, respectively. The polarization of the photons coming on the crossing points of the two rings cannot be distinguished, and therefore the paired photon state is polarization-entangled as represented by $(|HV\rangle +|VH\rangle )/\sqrt{2}$, where |*H*〉 and |*V*〉 indicate the horizontally and vertically linearly polarized photon state, respectively. A series of two irises are placed on each crossing point behind the band pass filter to extract the paired photons that are entangled but emitted in two directions, and the photons are separately coupled into two multimode fibers whose core diameter is 50 *μ*m. Here we call the photon path through the right (left) crossing point of the two photon rings as Path A (B).

In order to confirm the polarization correlation of the paired photons we executed a coincidence measurement between the optical fiber outputs. Each output is detected by an avalanche photodiode mounted on a single photon counting module (SPCM). The SPCM signal, synchronized with repeating pulses of the Ti:sapphire laser, is counted using a PCI board (Time Harp 200, Pico Quanta). To selectively pick up the coincidence signals, a logical AND is performed on the two different SPCM signals or two optical fiber outputs. From the polarization correlation measurement taken on the coincidence photons (see SI), we confirmed that the detection rate of the entangled photon pairs is 14 to 16 kHz.

### GaAs lateral quantum dot

**Figure 2:**
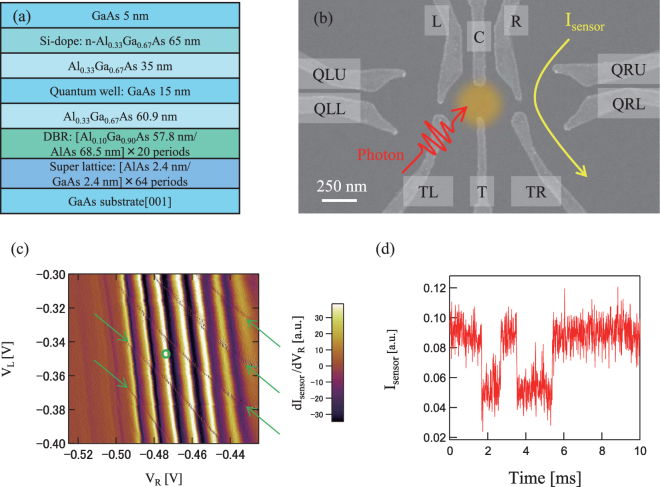
QD formation in the GaAs/AlGaAs heterostructure QW. (**a**) Layer profile of the quantum well wafer used for fabricating the QD device. A 15 nm GaAs well is sandwiched by two thick barrier layers of Al_0.33_Ga_0.67_As. Below the barrier layer a DBR structure consisting of periodic Al_0.10_Ga_0.90_As/AlAs quarter-wave stacks is embedded. (**b**) Scanning electron micrograph of a test device fabricated in the same way. The dot position formed by surface gates is pictorially shown by the orange circle. On the right side of the dot, an additional dot is formed as a charge sensor and its conduction is measured by a RF circuit. (**c**) Typical stability diagram of the single dot characterized by the differential conductance of the charge sensor current, dI_sensor_/dV_R_ as a function of two gate voltages V_L_ and V_R_. The thin gray lines indicated by the green arrows are the charge transition lines of the photo-electron trapping QD. We set the gate voltages V_L_ and V_R_ at the green circle position between the charge transition lines for the photon irradiation experiment. (**d**) Typical time trace of the single electron tunnelling between the dot and source-drain electrodes. When the electron enters (leaves) the dot, I_sensor_ abruptly drops (rises). The time trace is taken over 10 *μ*sec for integrating the I_sensor_ data.

The GaAs QD device used for trapping single photons is defined by surface Schottky gates in a two-dimensional electron gas formed in a 15 nm thick GaAs well located between two thick Al_0.33_Ga_0.67_As barriers (see Fig. 2(a,b))^29,30^. To enhance the efficiency of photon absorption in the dot, a distributed Bragg reflector (DBR) consisted of multiple Al_0.10_Ga_0.90_As/AlAs bi-layers is embedded. For the photon trapping experiment a single QD is formed by applying appropriate negative voltages to the gates to deplete the carrier density underneath them and in their direct surrounding. On both side of the QD, additional dots are placed as charge sensors^31,32^, but only the right charge sensor is used in the experiment here. In addition a 300 nm thick Au/Ti mask (not seen in Fig. 2(b)) having a 500 nm-diameter aperture is placed on top of the photon-electron trapping QD to avoid photon irradiation on the device area outside the dot. All measurements are carried out for the dot device placed in a dilution refrigerator (TRITON 200) at a base temperature of 25 mK. The photons are irradiated on the dot through an optical window equipped at the bottom of the fridge. The single photo-generated electron is detected by measuring the QD charge sensor response upon the photon irradiation. A change in the number of electrons in the dot modifies the charge sensor current, I_sensor_, which is measured using a radio frequency reflectometry technique, which we call: rf-QD^33^. We set the QD sample into a RF-circuit which shows resonance at 215.5 MHz. The minimal charge detection time achieved is 10 *μ*sec limited by the time of integration of I_sensor_ needed to keep the signal-to-noise ratio 10 × log_10_(I_sensor_signal_/I_sensor_noise_) higher than 0 dB. Note that the RF-circuit response time is much less than 10 *μ*sec.

The QD charge state as a function of gate voltages can be determined using the charge sensor as shown in Fig. 2(c). Differential conductance dI_sensor_/dV_R_ of the charge sensor versus two gate voltages V_R_ and V_L_ is shown without the photon irradiation. Sharp lines indicated by arrows are the charge state transition lines separated by the sum of the Coulomb gap and the QD level spacing. The lowest line indicates the transition between the zero and one electron state in the dot, and the number of electrons in the dot increases one-by-one every time when crossing the transition line upward. Figure 2(d) shows the real time trace of I_sensor_ measured on one of the transition lines. Transitions between the two current values indicate the charge tunnelling between the dot and the source or drain electrode. The time scale of electron trapping can be evaluated from the time interval needed for I_sensor_ switching from the lower level of about 0.05 to the higher level of about 0.09. Note that value of I_sensor_ depends on the gate bias position of the charge sensor because the charge sensor conductance shows Coulomb oscillations as seen in the background in Fig. 2(c).

In the photon irradiation experiment we adjusted the electron trapping time to a few hundred micro seconds which is about ten times longer than the averaged interval of the down converted photons. The charge state is tuned to be in the Coulomb gap or roughly 0.5 meV below the Fermi energy of the contact leads (green circle in Fig. 2(c))^34^. This energy is much larger than the electron temperature of 100 mK in the contact leads.

The mechanism of photo-electron trapping in this experiment is explained in detail in ref.^26^. Photo-electrons are created in the dot by optical excitation of electron-hole pairs in the GaAs QW at the Γ-point. The dot is confined laterally by a electro-static potential and vertically by the AlGaAs/GaAs/AlGaAs double heterostructure, so that the photo-generated electron remains in the dot being trapped by the lateral potential whereas the hole laterally escapes from the dot.

## Results

### Coincidence measurement on the electron and the photon

Next, we generate photon pairs and irradiate one of the paired photons on the dot in Path A and the other on SPCM in Path B. A photo-electron is generated in the dot and detected by the charge sensor as shown in Fig. 2(d), leaving the photon detection setup of Path B unchanged. The charge sensor signal, I_sensor_ and the photon counter signal, V_photon_ are both monitored using a digitizer whose data acquisition rate is 100 MHz. The integration time of I_sensor_, and V_photon_ is 10 *μ*sec, and 10 nsec, respectively.

In order to obtain the photo-electron trapping efficiency *η*_QD_ we separately performed a single pulse excitation experiment. We first evaluated the light transmission through the metal mask aperture above the dot (described before). On the other hand, the laser beam spot size on the metal mask is approximately 10 *μ*m. Thus we could derive the transmission coefficient T_aper_ of about 0.25% although it is difficult to discuss sub-wavelength hole transmission coefficient^35^.

In addition, we tune pulsed laser intensity by using several neutral density filters such that a few to a few tens of photons go through the optical mask on top of the dot in each pulse irradiation. A single light pulse is picked up from the pulse train with an acoustic optical modulator(AOM) which is triggered by an external logical signal. We count the photo-electron trapping events which are detected synchronously detected by the charge sensor with the laser pulsing on the QD. Finally *η*_QD_ is obtained by calculation using the following equation.

1 $${\eta }_{{\rm{QD}}}=\frac{({\rm{the}}\,{\rm{number}}\,{\rm{of}}\,{\rm{photo}}-{\rm{electron}}\,{\rm{trapping}}\,{\rm{events}})}{({\rm{the}}\,{\rm{number}}\,{\rm{of}}\,{\rm{pulse}}\,{\rm{irradiations}})\times ({\rm{photon}}\,{\rm{number}}\,{\rm{in}}\,{\rm{a}}\,{\rm{single}}\,{\rm{pulse}})\times {{\rm{T}}}_{{\rm{aper}}}}$$

**Figure 3:**
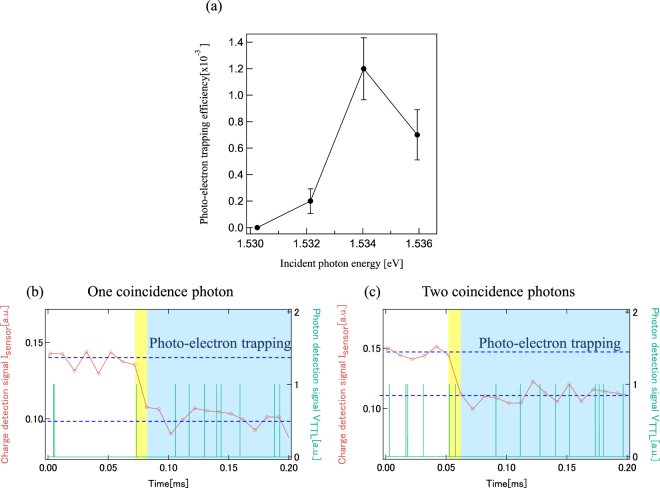
Quantum efficiency of the QD device and real-time measurement of a single photo-electron-photon pair. (**a**) Energy spectrum of photo-electron trapping efficiency on the QD taken under external in-plane magnetic field of 9 T. The horizontal axis is energy of incident photon. The peak at 1.534 eV corresponds to resonant excitation of the heavy hole exciton. (**b**,**c**) Examples of simultaneously measured time traces for the charge sensor current I_sensor_ in red and the SPCM photon detection signal V_photon_ in green. One photon and two photons are detected by the SPCM in the 10 *μ*sec photon-electron trapping time window in yellow of photon trapping by the dot in (**b**,**c**), respectively. The I_sensor_ falls indicate that one photo-electron is trapped by the dot at some point in the time window.

The efficiency of photo-electron trapping by the dot depends on the incident photon energy on the dot as shown in Fig. 3(a). The efficiency is evaluated for the incident photons which pass through the optical mask above the dot and is contained in a single light pulse (see SI). The efficiency is maximum of 0.15% for the resonant excitation of heavy hole excitons at 1.534 eV. We additionally measure photo-luminescence spectrum(PL) on the same QW wafer at 77 K. The resonance energy of the HH exciton as shown in Fig. 3(a) is consistent with the PL spectrum (see SI). Besides, we previously experimentally verified HH and LH resonant excitation on GaAs lateral QDs^26^, and the resonance peak in Fig. 3(a) is similar to the reported result. In the following experiment, we set the laser photon energy (1.5345 eV) resonating with the HH excitons.

Figure 3(b,c) show the time traces of I_sensor_ and V_photon_ measured simultaneously: red circles for I_sensor_ and green spikes for V_photon_. Each V_photon_ spike indicates a single photon detection by the single photon counter. The I_sensor_ time trace measured upon the photo-electron trapping is similar to that of Fig. 2(d). I_sensor_ abruptly decreases to the low level upon photon irradiation and then stays at the low level, indicating that a single photo-electron is trapped by the dot in the 10 *μ*sec time window of the yellow color and stays in the dot. In most cases one, or two V_photon_ spikes are detected at the SPCM in this time window as shown in (b), or (c), respectively. We repeated the experiments of separately irradiating paired photons on the dot and on the photon counter, and finally acquired 27 traces indicating the photo-electron trapping by the dot. We calculated the probability P_coinci_(N) of finding N (=0, 1, 2, …) photons in the 10 *μ*sec trapping time window conditioned on single photo-electron detection at the dot. The obtained probabilities are P_coinci_(0) = 44%, P_coinci_(1) = 41%, and P_coinci_(2) = 15%. We estimate possible errors of P_coinci_(N) by assuming that the errors of the probabilities follow Gaussian distribution. The evaluated errors of P_coinci_(N) are 6.8 to 9.7% and 0.947 to 1.62%, respectively (See SI, Table SI1).

To examine the effect of dark counts in the charge sensing o the observed photo-electron trapping events we irradiated the SPDC photons on the outside of the optical mask aperture. Then we observed no trapping events for 1 hour time trace acquisition. Note that we observe on or two trapping events on average for the 10 sec time trace acquisition when the irradiating position is on the aperture. Therefore, we confirmed that the dark count effect can be neglected in the present setup for the QD charge state being deep in the Coulomb gap (see Fig. 2(c)).

Concerning the photon detector, the dark count rate is typically 50 Hz or at most 100 Hz, which is much less than the SPDC photon detection rate of a few ten to one hundred kHz. Therefore, we assume that the dark count effect can be neglected also for the photon detection.

To confirm if the obtained photo-electron trapping events are induced by SPDC photons or dark count, we irradiate the SPDC photons to outside the aperture on the optical mask. In a case that the irradiating position is on the aperture, 1 or 2 trapping events are observed during 10 sec irradiation. Otherwise, we do not see any trapping events for 1 hour time trace acquisition. The result indicates that when the QD is tuned in Coulomb blockade regime substantial influence from the dark counting in the charge sensor like accidental or spontaneous charging is completely suppressed while acquiring the simultaneous time traces.

Besides, dark counting rate of the photon detector is typically 50 Hz and at most 100 Hz. Because the rate is much less than SPDC photon detection rate of a few ten to one hundred kHz, we think that contribution of the dark counting on the photon detector to the result can also be ignored.

## Discussion

### Probability of a single electron-photon pair creation

**Figure 4:**
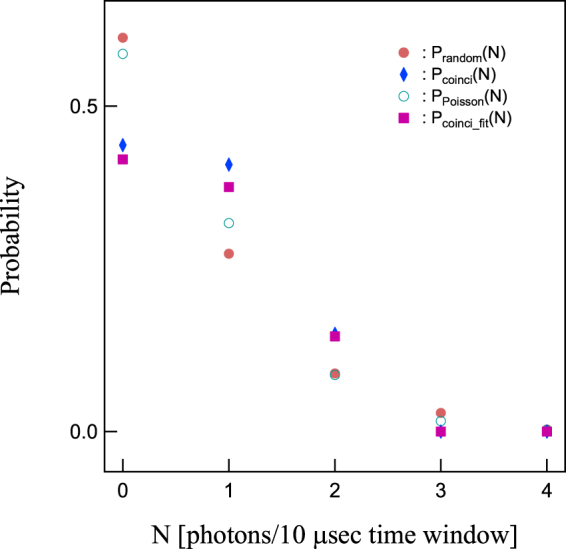
Comparison between P_coinci_(N) and P_random_(N), and best fits P_coinci_fit_(N) to P_coinci_(N) with P_el–ph_ as a parameter. The N photon detection probability P_Posioon_(N) (green circles) calculated for a Poisson distribution with a 55 kHz average photon detection rate well reproduces P_random_(N) (orange circles). The least squared fits P_coinci_fit_(N) (purple squares) to P_coinci_(N) (blue diamonds) is obtained using P_el–ph_ as a parameter. The statistics of P_coinci_(N) and P_coinci_fit_(N) are significantly deviated from those of P_random_(N) and P_Poisson_(N), originating from creation of single pairs of a photo-electron and a photon.

In Fig. 3(b) two photons are detected in the photo-electron trapping window. This occurs unintentionally because the time window is not small enough compared to the average interval of the photon counting signals. Note that the SPCM photon detection observed in the photo-electron trapping time window does not always indicate that the photo-electron in the dot detected by the charge sensor and the photon detected by SPCM are coincident or originated from the same SPDC photon pair because the generation efficiency of the entangled photon pairs is relatively low. To evaluate the probability of the non-coincident detection in P_coinci_(1) and P_coinci_(2) we derived the averaged probability P_random_(N) of detecting N = 0 to 2 photons per 10 *μ*sec by the photon counter regardless of whether the photo-electron is detected or not. The obtained P_random_(0) = 60.5%, P_random_(1) = 27% and P_random_(2) = 8.9%, respectively. Note that the photon counting rate for Path B measured just before the coincidence experiment is about 55 kHz. We calculate the probability P_Poisson_(N) of the photon detection in the 10 *μ*sec time window using the Poisson statistics and find good agreement with P_random_(N) for N = 0 to 4 (See Fig. 4). Compared with these values of P_random_(1) and P_random_(2), P_coinci_(1) and P_coinci_(2) are significantly larger, but P_coinci_(0) is significantly smaller than P_random_(0), because of the contribution from the true coincident detection of a photon and a photo-electron generated from the same SPDC photon pair.

We estimate a conditional probability P_el–ph_ that a truly correlated photon is detected given that a photo-electron is detected by the QD charge sensor, by comparing P_coinci_(N) and P_random_(N). P_coinci_(N) and P_random_(0) directly related to each other with P_el–ph_ as a parameter:

2 $${{\rm{P}}}_{{\rm{coinci}}}\mathrm{(0)}=\mathrm{(1}-{{\rm{P}}}_{{\rm{el}}-{\rm{ph}}})\times {{\rm{P}}}_{{\rm{random}}}\mathrm{(0)}$$

3 $${{\rm{P}}}_{{\rm{coinci}}}({\rm{N}})={{\rm{P}}}_{{\rm{el}}-{\rm{ph}}}\times {{\rm{P}}}_{{\rm{random}}}({\rm{N}}-\mathrm{1)}+\mathrm{(1}-{{\rm{P}}}_{{\rm{el}}-{\rm{ph}}})\times {{\rm{P}}}_{{\rm{random}}}({\rm{N}})\,({\rm{For}}\,{\rm{the}}\,{\rm{case}}\,{\rm{of}}\,1\,\leqq \,{\rm{N}}\,\leqq \,\mathrm{4)}$$

We use least squares method to fit these functions to the measured P_coinci_(N) to evaluate P_el–ph_. The adapted values P_coinci_fit_(N) for P_coinci_(N) with N = 0 to 4 by optimizing P_el–ph_ are shown by te purple symbols in Fig. 4. The best agreement between P_coinci_fit_(N) and P_coinci_(N) is obtained with P_el–ph_ of 31%. This value is comparable to a probability of a paired photon detection evaluated at coincidence measurement of paired photons (See SI).

### Further consideration about the result

By taking into account P_el–ph_, we can evaluate the conversion efficiency from SHG to SPDC. The SHG average power P_SHG_ is roughly 100 mW and with the wavelength *λ*_SHG_ of 404 nm or the photon energy E_SHG_ of 3.0681 eV in energy. We use these values to calculate the SHG photon number Γ_SHG_ in 1 second using the following equation:

4 $$\begin{array}{rcl}{{\rm{\Gamma }}}_{{\rm{SHG}}} & = & \frac{{{\rm{P}}}_{{\rm{SHG}}}}{{{\rm{E}}}_{{\rm{SHG}}}\times 1.602\times {10}^{-19}}\end{array}$$

5 $$\begin{array}{rcl}\quad \quad  & \simeq  & 2.0\times {10}^{17}\,[{\rm{photons}}/\sec ],\end{array}$$

where the factor of 1.602 × 10^−19^ is the scaling ratio between electron bolt and Joule.

The conversion efficiency *ξ*_conv_ from photon pair to electron-photon pair obtained here is mainly limited by the QD photon absorption coefficient *η*_QD_ of 1.2 × 10^−3^ (discussed before) and estimated by multiplying *η*_QD_ by the SPCM detection efficiency *η*_SPCM_ which is about 50% at 800 nm photon wavelength. Therefore we obtain ${\xi }_{{\rm{conv}}}\simeq 6.0\times {10}^{-4}$.

Finally, we consider ways to improve the coincidence probability. Recent technical advances in rf-QDs charge sensing enable us to shorten the charge detection time down to 100 nsec^36^. If the charge detection time decreased to 1 *μ*sec, one photon detection probability in a 1 *μ*sec time window P_random_(1) can be suppressed down to 1/10 of the current value, while the probability of the true coincident events is unchanged. For the case of 55 kHz average photon detection rate and the 1 *μ*sec time window, P_random_(0) = 95%, P_random_(1) = 5.2% and ${{\rm{P}}}_{{\rm{random}}}({\rm{N}}\,\geqq \,\mathrm{2)}$ is less than 1%. This shortening of the time window indicates that probability of detecting only a truly correlated photon given that a photo-electron is detected can be enhanced. To increase the generation rate of the polarization-entangled photon pairs will also be efficient to raise the probability of finding the true coincident pairs of the photo-electron and the photon.

## Summary

In summary, we performed the coincidence measurement of single photo-electrons in a quantum dot and single photons using a down converted polarization-entangled photon pair. We compared the probability of detecting the photon by the photon counter between with and without the photo-electron detection in the dot, and found the former to be substantially higher than the latter. From this comparison we estimated the coincidence rate to be comparable to the emission rate of the original photon pairs. We previously demonstrated that single electron spins are photo-generated in QDs by irradiating single photons with preserving the angular momentum. Consequently combining with the present result, we here presume that the angular momentum correlation is preserved between the single photo-electrons in the dot and the single photons that are spatially separated but generated from the same polarization-entangled photon pairs.

## Methods

### Device fabrication

Our device is fabricated from a GaAs/AlGaAs heterostructure wafer which defines a 2 DEG. The electron density and mobility in the 2 DEG are 1.91 × 10^11^ cm^−2^ and 1.24 × 10^6^ cm^2^/V · sec at 4.2 K, respectively. Surface Schottky fine gates of Au/Ti to define the QD are fabricated using electron beam lithography as shown in Fig. 2(b). The fine gates are covered with a 120 nm thick insulator layer of calixarene and a 300 nm thick Au/Ti mask, having a 500 nm-diameter aperture which is placed on top of the photon-trapping QD.

## Electronic supplementary material

Supplementary Information
