# Supplementary material for: Single electron-photon pair creation from a single polarization-entangled photon pair
Source: Sci Rep. 2017 Dec 5;7:16968. doi: 10.1038/s41598-017-16899-w (PMC5717106; doi:10.1038/s41598-017-16899-w)
Supplement: Supplementary file 1 — Supplementary Information [file 41598_2017_16899_MOESM1_ESM.pdf]

Single electron-photon pair creation from a single polarization-entangled photon pair

Kazuyuki Kuroyama,[1,*] Marcus Larsson,[1] Sadashige Matsuo,[1] Takafumi Fujita,[1] Sascha R. Valentin,[2] Arne Ludwig,[2] Andreas D. Wieck,[2] Akira Oiwa,[3] and Seigo Tarucha[1,4,†]

[1] *Department of Applied Physics, The University of Tokyo, Bunkyo-ku, Tokyo, Japan*
[2] *Lehrstuhl für Angewandte Festkörperphysik, Ruhr-Universität, Bochum, Germany*
[3] *The Institute of Scientific and Industrial Research, Osaka University, Ibaraki, Osaka, Japan*
[4] *Center for Emergent Materials Science, RIKEN, Wako, Saitama, Japan* [†]

[*] kuroyama@meso.t.u-tokyo.ac.jp
[†] tarucha@ap.t.u-tokyo.ac.jp

SUPPLEMENTARY NOTE 1: VISIBILITY OF POLARIZATION CORRELATION

In order to confirm the polarization correlation and its visibility on the coincidence photons, we measured the polarization states of $|H_A V_B\rangle$ and $|V_A H_B\rangle$. The measurement setup is shown in Fig.SI1(a). A polarization beam splitter (PBS) is placed in Path A and Path B in order to project the photon polarization in the linear polarization basis. A half wave plate (HWP) is inserted in Path A to observe the visibility of the polarization correlation. The coincidence count of the photon signals detected in the two paths is extracted using a logical AND circuit. Results of the projection measurement onto the $|H_A H_B\rangle$ (red curve) and $|H_A V_B\rangle$ (blue curve) states are shown in Fig.SI1(b). In the $|H_A H_B\rangle$ projection the coincidence count is maximal when the rotating angle of polarization on Path A is 90 degrees, indicating that the paired photons have the $|V_A H_B\rangle$ state. On the other hand, in the $|H_A V_B\rangle$ projection it is maximal for the rotating angle of 180 degrees, indicating that the paired photons have the $|H_A V_B\rangle$ state. The visibility of the polarization correlation for the coincident photon pairs obtained in this experiment is 96 %. In the correlation measurement, probability of coincident photon detection with respect to photon detection at one of the photon counters is calculated at most about 27 %.

SUPPLEMENTARY NOTE 2: RANDOM SAMPLING AND PHOTON STATISTICS

In the random sampling, N(=0, 1, 2,...) photons are observed in the 10 μsec time window (See Fig.SI2). This measurement is repeated for a number of times to derive the probability $\mathrm{P_{random}}$(N) of detecting N photons independent of whether the photo-electron is detected in the dot or not. Figure SI2 shows an example of the random sampling of $\mathrm{V_{photon}}$ as a function of time in a 0.2 msec time window. The averaged $\mathrm{P_{random}}$(N) in the 10 μsec time window is shown by the solid circles in Fig.4. $\mathrm{P_{random}}$(N) progressively decreases with increasing N from 0 to 2 and are $\simeq$ 0 for N$\geqq$3. As for the down converted photons their photon statistics should follow the Poisson distribution. Since the 76 MHz repetition rate pulsed laser is used for generating SPDC photons, the probabilities of detecting N photons in the 10 μsec time window can be calculated using a binomial distribution. Particularly for the case of a large number of the $\mathrm{V_{photon}}$ pulses included in the time window the probability $\mathrm{P_{random}}$(N) approximately follows the Poisson distribution. The calculated $\mathrm{P_{random}}$(N) is shown by the open circles in Fig.4. We see good agreement between the calculation and experiment. Here the Poisson distribution is constructed using the photon detection rate of 55 kHz which is measured just before executing the coincidence measurement.

SUPPLEMENTARY NOTE 3: HH RESONANCE PEAK

To confirm that the peak observed in Fig.3(a) is due to the HH excitation, we investigated the PL spectrum of the QW wafer measured at 77 K (Fig.S3). The PL peak is asymmetric due to contributions from the light hole (LH) excitation because both states of HH and LH are populated at this temperature. We fitted two Voigt functions to the PL spectrum as indicated by the blue curve, and decomposed the asymmetric peak into two resonance peaks HH, and LH, at 1.5276 eV and 1.5341 eV, respectively. By taking into account teh temperature dependence of GaAs band gap energy [1], we evaluated the HH resonant excitation energy of 1.5357 eV, which is close to the peak position in Fig.3(a).

References

[1] Varshni, Y. P. Temperature dependence of the energy gap in semiconductors. *Physica*, 34:149–154, 1967. doi:10.1016/0031-8914(67)90062-6.

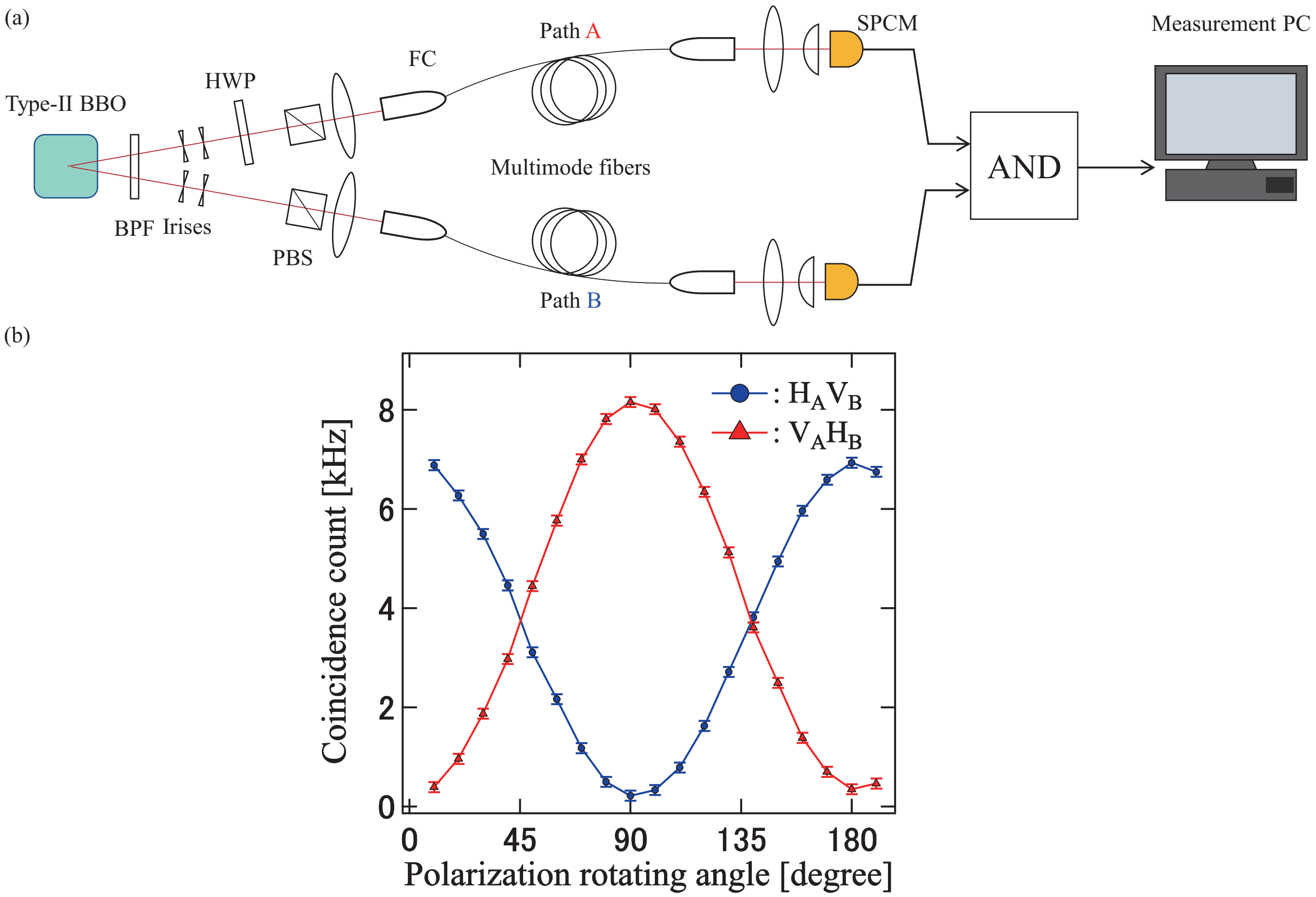

FIG. 1. *

Figure SI1 | Polarization correlation measurement on down converted photon pairs.(**a**) Schematic of the optical setup for the polarization correlation measurement taken on the down converted photons. The polarization state of the photon in path A is rotated by passing through the HWP. PBSs are used to take the projection measurement onto the $|H_AH_B\rangle$ or $|H_AV_B\rangle$ state. We take a logical AND on the photon detection signals coming from the different SPCMs to extract the coincidence photons. (**b**) Polarization correlation measured on the simultaneously detected paired photons. The red curve and the blue one are taken by the projection measurement on the $|H_AH_B\rangle$ state and on the $|H_AV_B\rangle$ state, respectively. For the $|H_AH_B\rangle$ projection when the polarization of path A is rotated by 90 degree, (i.e. the projection is done onto the $|V_AH_B\rangle$ state) the coincidence count is maximal. On the other hand, for the $|H_AV_B\rangle$ projection, when the polarization is rotated by 180 degree (i.e. the projection is done onto the $|H_AV_B\rangle$, the original state), maximal coincidence is obtained.

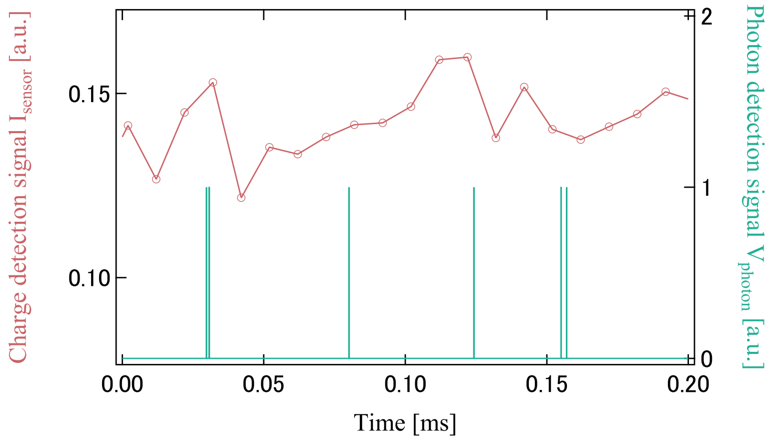

FIG. 2. *

Figure SI2 | Random sampling. An example time trace of the random samplings taken while the electron trapping does not occur. We count the number of the detected photons for every 10 μsec time window of the charge sensor. In this time trace, one or two photons are observed in 10 μsec.

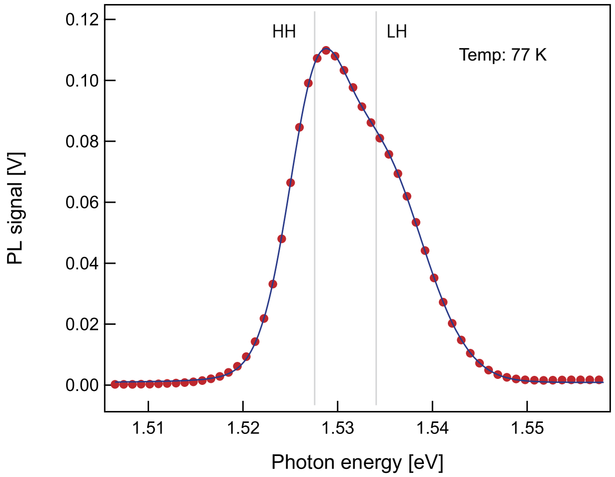

FIG. 3. *

Figure SI3 | PL spectrum at 77 K.PL spectrum of the QW wafer measured at 77 k.The experimental data are shown by the red circles, while the best fit to the data with sum of two Voigt functions indicated by the blue line. The PL peak is decomposed into two Voigt functions corresponding to the HH and LH peaks where positions are indicated by the thin vertical lines at 1.5726 eV, and 1.5341 eV, respectively.

The number of photons detected in the time window	N=0	N=1	N=2
one electron creation : $P_{coinci}(N)$	44±9.7 %	41±9.5 %	15±6.8%
no electron creation : $P_{random}(N)$	60.5±1.62 %	27.3±1.48 %	8.93±0.947 %

TABLE I. *

Table SI1 | Enhancement of the photon detection probability due to the electron creation. The upper column shows the probability of finding N (=0, 1, 2) photons in the photon-electron trapping time window of 10 μsec. The lower is an averaged probability of detecting N=0, 1, 2 photons per 10 μsec by the photon counter, regardless of whether the photo-electron is detected or not. The single photon detection twice in the 10 μsec occurs unintentionally because the 10 μsec is not short enough compared to the average interval duration of the photon detection signals. $P_{coinci}(1)$ and $P_{coinci}(2)$ are both significantly greater than $P_{random}(1)$ and $P_{random}(2)$ due to the true coincident detection of the photon and the photo-electron.
